# The Human Splice Variant Δ16HER2 Induces Rapid Tumor Onset in a Reporter Transgenic Mouse

**DOI:** 10.1371/journal.pone.0018727

**Published:** 2011-04-29

**Authors:** Cristina Marchini, Federico Gabrielli, Manuela Iezzi, Santa Zenobi, Maura Montani, Lucia Pietrella, Cristina Kalogris, Anna Rossini, Valentina Ciravolo, Lorenzo Castagnoli, Elda Tagliabue, Serenella M. Pupa, Piero Musiani, Paolo Monaci, Sylvie Menard, Augusto Amici

**Affiliations:** 1 Department of Bioscience and Biotechnology, University of Camerino, Camerino, Italy; 2 Aging Research Centre, G. d'Annunzio University, Chieti, Italy; 3 Molecular Targeting Unit, Department of Experimental Oncology and Molecular Medicine, Fondazione IRCCS, Istituto Nazionale dei Tumori, AmadeoLab, Milan, Italy; University of California, Los Angeles, and Cedars-Sinai Medical Center, United States of America

## Abstract

Several transgenic mice models solidly support the hypothesis that HER2 (ERBB2) overexpression or mutation promotes tumorigenesis. Recently, a HER2 splice variant lacking exon-16 (Δ16HER2) has been detected in human breast carcinomas. This alternative protein, a normal byproduct of HER2, has an increased transforming potency compared to wild-type (wt) HER2 receptors. To examine the ability of Δ16HER2 to transform mammary epithelium *in vivo* and to monitor Δ16HER2-driven tumorigenesis in live mice, we generated and characterized a mouse line that transgenically expresses both human Δ16HER2 and firefly luciferase under the transcriptional control of the MMTV promoter. All the transgenic females developed multifocal mammary tumors with a rapid onset and an average latency of 15.11 weeks. Immunohistochemical analysis revealed the concurrent expression of luciferase and the human Δ16HER2 oncogene only in the mammary gland and in strict correlation with tumor development. Transgenic Δ16HER2 expressed on the tumor cell plasma membrane from spontaneous mammary adenocarcinomas formed constitutively active homodimers able to activate the oncogenic signal transduction pathway mediated through Src kinase. These new transgenic animals demonstrate the ability of the human Δ16HER2 isoform to transform “per se” mammary epithelium *in vivo*. The high tumor incidence as well as the short latency strongly suggests that the Δ16HER2 splice variant represents the transforming form of the HER2 oncoprotein.

## Introduction

Numerous studies estimate that the oncogene HER2 is overexpressed in 20–30% of primary breast cancers and this alteration correlates with poor prognosis [1]. Further support for the involvement of HER2 in the initiation and progression of breast cancer comes from analysis of transgenic mice generated by targeting overexpression of activated neu (the rat homolog of HER2) to the mammary gland under the control of the murine mammary tumor virus (MMTV) promoter [2]. Activated neu is a mutated form with valine instead of glutamic acid at residue 664 in the transmembrane domain. Although rapid onset (11–13 weeks) of multifocal mammary tumors was observed in the majority of activated neu transgenic mice, this mutation has never been observed in human cancers, which only present amplification of the HER2 gene copy number and consequent overexpression of HER2 protein on the cell membrane. In wt neu-expressing mice under the MMTV promoter, focal mammary tumors arise next to hyperplastic mammary tissue after a long latency period (17–48 weeks) [3], suggesting that genetic alterations in addition to that inducing HER2 overexpression are required for mammary transformation. Notably, tumors in these transgenic mice arose only when the oncoprotein carried mutations involving small deletions in the extracellular domain that promote HER2/neu transforming activity through formation of intermolecular disulfide bonds [4]. More recently, transgenic mice were generated with wt human HER2 under the whey acidic protein promoter; however while HER2 was expressed in the mammary glands, no mammary neoplastic transformation was ever detected in any animal [5]. Another human wt HER2 transgenic model under the direction of MMTV developed human HER2-overexpressing breast tumors but with a long latency of about 28.6 weeks [6]. Sequencing of the human HER2 transcripts from primary mammary tumors that developed in the transgenic founder mouse revealed an in-frame 15-bp deletion in the wt HER2 juxtamembrane region potentially affecting cysteine-mediated dimerization. In this context, recent studies have reported that overexpression of HER2 alone does not seem sufficient to generate mammary tumors in mice and requires activating mutations that affect the number of cysteines to become oncogenic [7]. Indeed, it has been reported that expression of wt 611-carboxy-terminal fragments (CTF) in the mouse mammary gland led to the development of aggressive tumors [7], suggesting a causal role for CTF in tumorigenesis based on their ability to constitutively homodimerize.

Interestingly, an alternative splice form of human HER2 gene, Δ16HER2, containing an in-frame deletion in the same region mutated in neu or HER2 protooncogene transgenic mice, has been detected in human breast carcinomas [8], [9]. This deletion removes the relevant cysteine residues in HER2, disrupting the disulfide bond structure of the protein and leaving the remaining unpaired cysteine residues available for intermolecular bonding. For this reason, Δ16HER2 represents a constitutively active form similar to the mutated neu gene [8]. Δ16HER2 transcripts have been detected in a majority of breast tumors and reported to comprise 4–9% of total HER2 transcripts [9], [10], [11]. Moreover, this oncogenic isoform has been associated with trastuzumab resistance [10]. Transformation associated with HER2 overexpression might reflect the increase in absolute levels of this splice variant to a critical threshold for constitutive activation of HER2.

Here, we describe the generation and characterization of a new reporter transgenic mouse that expresses both the human Δ16HER2 splice isoform and the firefly luciferase under the transcriptional control of the MMTV promoter. Analyses of these genetically engineered mice demonstrate that Δ16HER2 constitutively homodimerizes on the tumor cell plasma membrane and is able to transform mammary epithelium *in vivo* through the oncogenic properties mediated by the downstream Src kinase signaling circuitry, making this splice variant a likely candidate for the transforming form of the HER2 oncoprotein.

## Results and Discussion

### Generation and characterization of the human Δ16HER2-LUC transgenic mice

**Figure 1:**
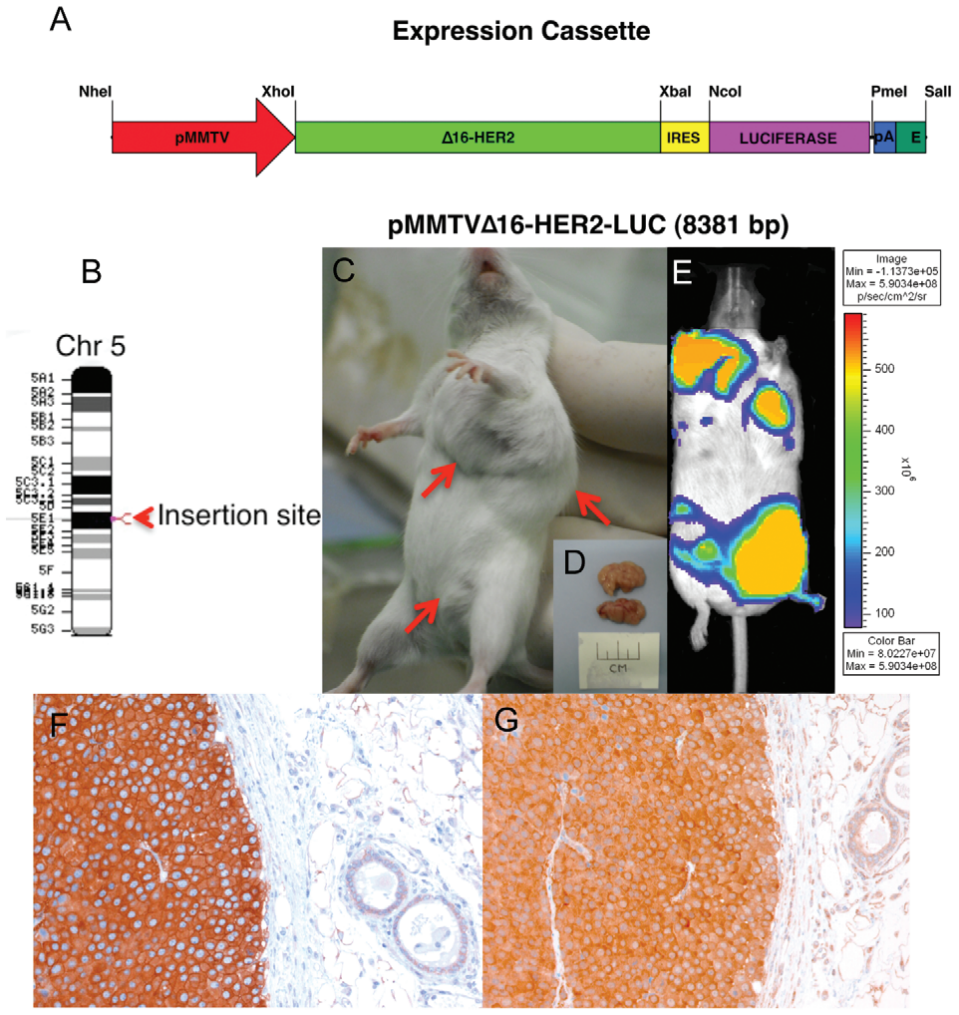
Δ16HER2-LUC transgenic mice develop spontaneous mammary tumors. (A) Schematic representation of the MMTV-driven human Δ16HER2-LUC transgene, with the MMTV LTR promoter (pMMTV, red), the human Δ16HER2 cDNA (green), the IRES (internal ribosome entry site, yellow), the luciferase cDNA (LUC, purple), and the termination signal from the SV40 (Poly A, blue). Relevant restriction sites are indicated. (B) Graphic representation of murine chromosome 5 divided into A–G regions and numbered subregions, showing that the transgene integrates in region E-1. (C, D) An F2 female transgenic mouse with primary breast tumors (arrows) just before tumor removal. (E) Bioluminescence analysis of a 28-week-old tumor-bearing Δ16HER2-LUC transgenic mouse (founder female), using the in VivoVision Systems, Xenogen. (F and G) Immunohistochemical detection of HER2 and luciferase, respectively, showing strong and uniform expression of both proteins in sections of a mammary tumor, while the normal duct on the right appears negative. Magnification: ×400.

We generated a Δ16HER2-LUC transgenic mouse using a bicistronic vector containing an IRES sequence between the human Δ16HER2 and the firefly luciferase genes to ensure their coordinated expression driven by the same MMTV promoter (Fig. 1A). Luciferase was chosen as a reporter since it is rapidly identified by optical imaging in live organisms and simultaneously allows accurate quantitation in tissue extracts and immunohistochemical detection with specific antibodies. Expression of luciferase and Δ16HER2 was verified on NIH3T3, HEK293 and MDAMB435 transfected cells before transgenic mouse generation (Fig. S1). The transgene was determined to have integrated at a single site, precisely at 85.72 Mb, on murine chromosome 5, region E-1 (NT109320.4), inside an intergenic region, NCBI Build m37.1 (Fig. 1B). BLAST analysis of this intergenic region revealed that it contains neither genes nor regulatory sequences. Transgene random insertion was found to have occurred exactly 1.17 Mb downstream of the non-histone chromosomal protein HMG-17-like gene and 718 Kb upstream of the centromere protein C1 gene. The great distance between the transgene and these predicted genes ensures that the insertion itself does not affect the tumorigenesis. Quantitative PCR analysis revealed a transgene copy number of 5. The founder female developed 8 spontaneous mammary tumors, starting at 18 weeks of age, and as expected, tumor localization was visualized in the live animal by bioluminescence analysis (Fig. 1E).

Because the MMTV promoter is hormonally regulated and tumor development in founder female might be enhanced by increased transgene expression in the mammary gland during pregnancy and lactation, we monitored the development of spontaneous mammary tumors by palpation in virgin females, starting from F2 generation of the transgenic line (see Materials and Methods). All transgenic females (n = 20) developed multiple asynchronous mammary tumors (4–5 tumors/mouse) between 12 and 19 weeks of age (Fig. S2A), with similar results in the F3 generation (n = 13). Transgenic virgin females, currently at the F4 generation, maintain that tumor onset schedule, with an average latency of 15.11±2.5 weeks (mean ± SD) (n = 35), indicating that the formation of Δ16HER2-overexpressing mammary tumors is a reproducible phenotype in Δ16HER2-LUC transgenic mice. Mammary tumors grew continuously, reaching 1–1.5 cm^3^ in a matter of weeks, as shown by mammary tumor growth curves (Fig. S2B). Sequencing of the transgenic HER2 transcript from mammary tumors confirmed the in-frame 48-bp deletion in the HER2 juxtamembranous region, eliminating amino acids 634 to 649. Of note, only a few transgene copies (only 5 copies were detected) are sufficient to drive neoplastic transformation of mammary epithelial cells in Δ16HER2-LUC mice, whereas 30–50 wtHER2 transgene copies are reportedly necessary to induce breast cancer in about 80% of MMTV-wtHER2 transgenic mice [6]. Since the Δ16HER2 splice variant represents about 10% of total HER2 transcript in human breast carcinoma, it is plausible that malignant transformation ensues when a critical threshold of Δ16HER2 is reached in mammary cells presenting HER2 gene amplification. Histologically, these fast-growing tumors were classified as invasive HER2-positive adenocarcinomas. Immunohistochemical analysis confirmed the concurrent expression of the human Δ16HER2 oncogene (Fig. 1F) and the luciferase gene (Fig. 1G) and revealed the specific staining for epithelial cells but not for stromal cells or adipocytes, although non-neoplastic mammary ducts were negative (Fig. 1F and G). Tumors consisted of cells with round nuclei and eosinophilic cytoplasm growing in solid sheets and packets traversed by delicate fibrovascular septa. Growth of these unencapsulated tumors compressed the surrounding tissues (Fig. 1F and G). Tumor-bearing transgenic females developed lung metastases beginning at 25 weeks of age (Fig. S3), suggesting particularly aggressive tumor behavior upon expression of the Δ16HER2 splice variant. The histological features of these pulmonary metastatic lesions were consistent with a primary breast tumor origin, with robust staining for HER2 and luciferase demonstrating high-level transgene expression (Fig. S3 middle and right panels, respectively). Although the MMTV promoter is noted for its targeted expression in mammary glands, its expression pattern in other tissues has been reported [6], [9], [12]. The tissue specificity of the transgene expression was verified by immunohistochemical analysis of the HER2 and luciferase expression pattern in mammary glands of Δ16HER2-LUC transgenic females (F2 generation) and also in other tissues, including brain, salivary glands, lymphoid compartment, reproductive organs, kidney, heart, lung, liver, stomach, intestine (Table 1). HER2 expression was detected only in the mammary gland and showed a strict correlation with tumor development. Although ovaries and uterus tissue sections showed a positive staining for HER2, they were negative for luciferase, suggesting that the transgene is not espressed in these tissues; the HER2 positivity might reflect cross-reactivity of anti-HER2 antibody with the murine HER2/neu protein. All tissues appeared architecturally normal, with invasive adenocarcinomas observed only in the mammary gland.

**Table 1:** Tissue-specific expression of the Δ16HER2-LUC transgene.

Organs	HER2	Luciferase
Liver	**−**	**−**
Kidney	**−**	**−**
Adrenal gland	**−**	**−**
Spleen	**−**	**−**
Lymph-nodes	**−**	**−**
Thymus	**−**	**−**
Stomach	**−**	**−**
Thyroid	**−**	**−**
Brain	**−**	**−**
Cerebellum	**−**	**−**
Salivary glands	**−**	**−**
Uterus	**+**	**−**
Trachea	**−**	**−**
Aorta	**−**	**−**
Heart	**−**	**−**
Lung	**−**	**−**
Eye	**−**	**−**
Ileum	**−**	**−**
Colon	**−**	**−**
Ovaries	**+**	**−**

### Δ16HER2 forms disulfide-bridged homodimers in Δ16HER2-LUC mammary tumors

**Figure 2:**
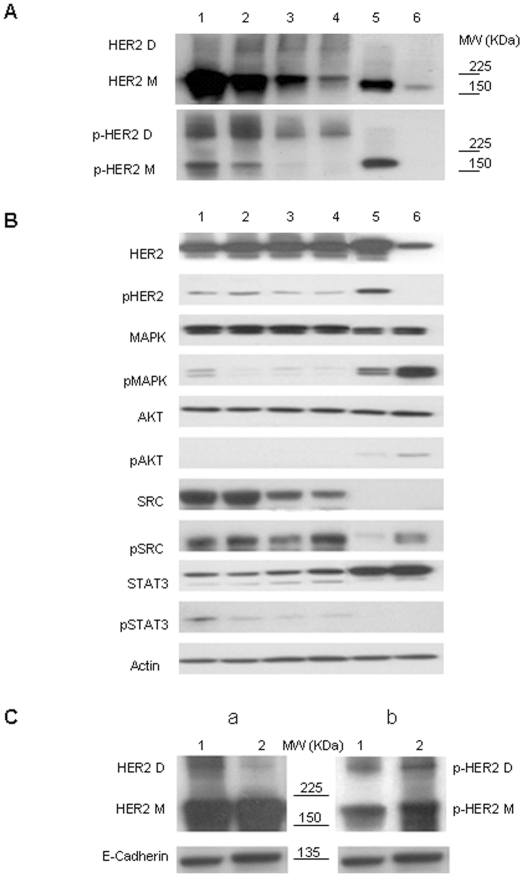
Western blot analysis of monomeric and dimeric Δ16HER2 forms and downstream signaling pathways. (A) Protein extracts from 4 different Δ16HER2 tumor samples (lanes 1–4) and breast cancer cell lines BT474 (lane 5) and ZR-75.1 (lane 6) were separated by 4–12% gradient SDS-PAGE under non-reducing conditions and probed, respectively, with MAb Ab3 (HER2 M and D) and PoAb anti-phospho-HER2 p-neu (p-HER2 M and D). (B) The same protein extracts (lanes 1–6) were analyzed under reducing conditions for activation of the following HER2 downstream oncogenic signaling pathways: MAPK, AKT, Src and STAT3. Actin was used to normalize protein loading. (C) Proteins from the pool of whole Δ16HER2 tumor extracts (lanes 1, subpanels a and b) and purified plasma membrane extracts (lanes 2, subpanels a and b) were separated by 4–12% gradient SDS-PAGE under non-reducing conditions and probed with MAb Ab3 (a) and PoAb antiphospho-HER2 p-neu (b). E-cadherin was used as a plasma membrane marker.

Western blot analysis of lysates of cells isolated *ex vivo* from 4 tumors obtained from 4 different mice revealed the presence of Δ16HER2 dimers migrating above 225 kDa (HER2 D) and the phosphorylation of Δ16HER2 D (p-HER2 D) in all samples under non-reducing conditions (Fig. 2A, lanes 1–4). The analysis of the plasma membrane component purified from the same Δ16HER2-positive tumor samples pooled to obtain an adequate amount of protein extracts revealed formation of stable, constitutively active Δ16HER2 homodimers on the tumor cell surface (Fig. 2C). Δ16HER2 D expression was less abundant than its monomeric counterpart both on whole lysates and plasma membrane extracts (Fig. 2C subpanel a, lanes 1 and 2, respectively), while both HER2 M and HER2 D were significantly more activated in the plasma membrane fraction than in crude tumor lysate (Fig. 2C subpanel b, lanes 2 and 1, respectively). E-cadherin was used as a plasma membrane marker. Analysis to determine whether Δ16HER2 expression leads to activation of oncogenic signal transduction pathways known to function downstream of constitutively activated HER2, such as mitogen-activated protein kinase (MAPK), AKT, Src kinase and STAT3, showed that, consistent with data reported in *in vitro* models [10], the oncogenic properties of Δ16HER2 were mediated through Src kinase (Fig. 2B, lanes 1–4). In our *in vivo* model, Src kinase activation was combined with that of STAT3, a downstream signal transducer in Src family kinase-mediated tumorigenesis [13]. Comparison of the signaling activity of Δ16HER2 with that of wtHER2 using BT474 (HER2-positive) and ZR75.1 (nearly HER2-negative) tumor extracts (Fig. 2A and B, lanes 5 and 6, respectively) revealed mainly activated MAPK- and AKT-mediated signaling circuitries. These findings suggest a mechanism through which the disulfide-bond homodimerized Δ16HER2 amplifies HER2 transforming potential, providing a crucial cell growth advantage.

### Bioluminescence analysis is predictive of tumor onset in human Δ16HER2-LUC transgenic mice

**Figure 3:**
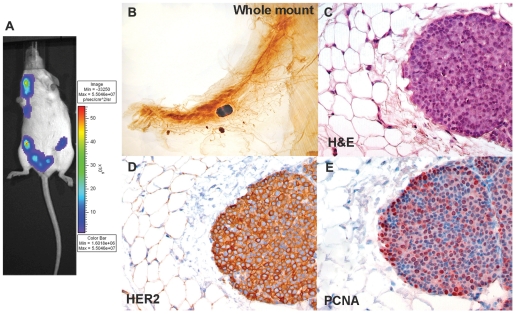
Detection of non-palpable tumors in Δ16HER2-LUC transgenic mice. (A) Bioluminescence analysis of an F2 female transgenic mouse at 14 weeks of age and (B) whole-mount analysis of the inguinal mammary glands; arrows indicate non-palpable tumors. (C) Hematoxylin-eosin and immunohistochemical staining for HER2 (D) and PCNA (E) of non-palpable mammary tumors at 14 weeks. Magnification: B, ×6; C–E, ×400.

Δ16HER2-LUC transgenic females were monitored for luciferase activity starting from 8 weeks of age. Bioluminescence analysis revealed luciferase activity in mammary glands of these mice one month before tumors became palpable (Fig. 3A), suggesting that luciferase reporter expression detected by such analysis might be predictive of tumor onset. Whole-mount (Fig. 3B) and histological (Fig. 3C–E) analysis of mammary glands confirmed the presence of small neoplastic masses, and immunohistochemical analysis of these non-palpable tumors revealed the expression of the HER2 protein (Fig. 3D). Whole-mount analysis of the mammary glands revealed 2 to 5 variously sized tumor masses arising preferentially from the ducts closest to the nipple. While large tumors displayed heterogeneous membrane staining for the human transgene, staining in the small tumors was homogeneously distributed in the tumor parenchyma. Finally, HER2 expression correlated with that of PCNA, which detects mitotic activity of transformed epithelial cells (Fig. 3E), suggesting that HER2 expression and hyperplasia are concomitant.

In conclusion, the high tumor incidence, the rapid tumor growth, as well as the significantly shorter latency period (15.11 vs 28.6 weeks) in our Δ16HER2 transgenic mice as compared with MMTV-wtHER2 transgenic mice described by Finkle et al. [6], strongly supports the candidacy of the Δ16HER2 splice variant as the transforming form of the HER2 oncoprotein. Indeed, the strong immunohistochemical staining for the transgene in tumor tissue, but not in non-neoplastic mammary ducts or in other tissues, supports the notion that expression of Δ16HER2 in the mammary gland is sufficient to induce malignant transformation in a single step. Conversely, support for the insufficiency of wtHER2 expression alone and without additional mutations for full malignant transformation comes from the study by Finkle et al. [6] who found that their transgenic mice developed mammary tumors in a stochastic manner and that wtHER2 expression was not confined to the tumor tissue but was also detected in morphologically normal mammary gland and in several other tissues. Indeed, those authors found sequence anomalies in the juxtamembrane region of wtHER2 in >80% of mammary tumors. The majority of these mutations affected the conserved cysteine residues and could function as a second hit in the transformation process in the female mammary gland [6].

The key role of the cysteine residues in the HER2 juxtamembrane region has been also demonstrated by Pedersen et al. [7], who showed that transgenic expression of a HER2 carboxy-terminal fragment (611-CTF) containing a transmembrane domain and a short extracellular region including the sequence deleted in Δ16HER2 led to the development of aggressive mammary tumors. The mechanism of activation of 611-CTF involved the formation of constitutive homodimers by intermolecular disulfide bonding [7], consistent with our present findings.

Studies to further evaluate the role of Δ16HER2 in tumor progression as well as its responsiveness to chemotherapy or targeted therapies for HER2-overexpressing breast carcinomas will benefit from the ability to noninvasively quantify tumor burden in live mice by measuring the activity of the reporter gene luciferase in our transgenic mouse model.

## Materials and Methods

### Generation and identification of transgenic mice

A novel bicistronic vector for *in vivo* examination of Δ16HER2 activity was designed. Each element of the expression cassette was sequentially cloned in the pGL3 control vector (Promega, USA) which contains the luciferase reporter gene under the control of SV40 promoter. This vector was modified and assembled with the following components: the MMTV promoter [2] cloned using XhoI e NheI restriction enzymes; IRES cloned using NcoI and XbaI restriction enzymes after elimination of the XbaI site downstream and insertion of a new XbaI site upstream of the luciferase gene; and Δ16HER2 cDNA cloned between MMTV promoter and IRES using XhoI and XbaI restriction enzymes and eliminating SV40 promoter. The plasmid obtained, pGL3-MMTV-Δ16HER2-IRES-LUC, was sequenced to verify the correct fragments insertion. To generate the reporter mouse for human Δ16HER2, the MMTV-Δ16HER2-IRES-LUC expression cassette (8381 bp) was isolated from the plasmid backbone by NheI and SalI digestions, purified and microinjected into fertilized eggs from FVB females (Facility of the Istituto San Raffaele, Milan, Italy). The FVB inbred mouse strain was used because of its known suitability for transgenic experimentation and genetic analysis [14]. Embryos were implanted into pseudopregnant CD1 surrogate mothers as described previously [15]. Founder mice (20 days of age) were shown to be transgenic by PCR using tail tissue lysates and primer sets specific for: MMTV promoter (forward 5′-CTAAATTCAGAAGTTAGAAATGGG-3′; reverse 5′-CCAAAACTTACTTAAGCCCTGG-3′), human HER2 (forward 5′-GTGCACCGGCACAGACATGA-3′; reverse 5′-TTGTTCAGCGGGTCTCCATT-3′) and luciferase (forward 5′-GGAAGACGCAAAAACATAAAGAAA-3′; reverse 5′-TGAGATGTGACGAACGTGTACAT-3′). From 37 live births, 3 positive female mice were identified (#6157, #6177 and #6180). In these three transgenic founders, the splice region of Δ16HER2 transgene was sequenced. Founder female #6157 developed 8 mammary tumors starting from 18 weeks of age, #6177 developed only 1 mammary tumor at 20 weeks of age, and #6180 died at 20 weeks of age due to the early development of multiple mammary tumors. Although founder #6180 was mated successfully and carried pups to term, all pups died within one day, most likely because this transgenic mother was unable to nurse them. The differences in tumor multiplicity and latency among transgenic lines generated with the same transgene construct might rest in the random integration of the transgene into the mouse genome, leading to different phenotypes and levels of transgene expression depending on the site of integration and on the number of transgene copies. The progeny from transgenic founder female #6157 was used for this study. In a breeding program to establish the #6157 transgenic line, an FVB non-transgenic male was mated with the transgenic founder female and the born pups were considered the F1 generation (founder×FVB). Sequencing of the Δ16HER2 transgene splice region was done in F1 mice. To expand the #6157 transgenic line, the transgenic male offspring of the F1 generation were mated with non-transgenic FVB females to obtain the F2 generation. Transgene distribution in subsequent offspring follows Mendelian rules. Routine screening of Δ16HER2-LUC transgenic mice was done using genomic DNA extracted from tail biopsies and genotyped by PCR analysis (primers: forward 5′-GGTCTGGACGTCCCAGTGTGA-3′; reverse 5′-GATAGAATGGCGCCGGCCCTT-3′). Transgenic females were monitored for mammary tumors by palpation and tumor measurements twice weekly. Progressively growing masses >1 mm mean diameters were considered as tumors and tumor volume was calculated as 0.5×d_1_^2^×d_2_, where d_1_ and d_2_ are the smaller and larger diameters, respectively.

Mice were treated according to the European Community guidelines. The Animal Research Committee of the University of Camerino authorized the experimental protocol.

### Determination of transgene insertion site and copy number

Transgene integration loci were examined using a restriction enzyme-PCR-based technique [16], [17] as described in Materials and Methods S1. Transgene copy number was determined using a method based on quantitative PCR [18].

### Cell Culture

Human breast carcinoma BT474 and ZR75.1 cells, which show HER2 oncoprotein overexpression (score 3+) and moderate expression (score 2+), respectively, and MDAMB435 cells, which score 0 for HER2 expression, were purchased from ATCC (Rockville, MD). Cells were maintained in RPMI 1640 medium (Sigma Chemical Co., St. Louis, MO) supplemented with L-glutamine and 10% fetal calf serum (FCS) (Sigma). Murine embryonic NIH3T3 fibroblasts was cultured in a 10% CO_2_ atmosphere, whereas human embryonic kidney HEK293 cells were cultured in a 5% CO_2_ atmosphere in RPMI 1640 medium (Sigma) supplemented with L-glutamine. Both lines were used together with MDAMB435 as recipient cells for transient transfection studies with pGL3-MMTV-Δ16HER2-LUC and control pGL3 vectors. Cells were routinely tested for mycoplasma contamination with a Mycoplasma Detection Kit (MycoAlert®, Lonza, Rockland, ME).

### Western blot analysis

Primary cultures from spontaneous tumors were lysed for 1 h on ice in lysis buffer (50 mM Tris-HCl pH 7.2, 150 mM NaCl, 100 mM NaF, 100 mM sodium pyruvate, 1% Triton X-100) containing protease inhibitors, 2 mM phenylmethylsulfonylfluoride, 10 µg/ml aprotinin, and 2 mM Na_3_VO_4_. Protein extracts were separated by electrophoresis on pre-cast polyacrylamide gels (Invitrogen, Carlsbad, CA) and transferred to hydrophobic polyvinylidene difluoride (PVDF) membranes (Amersham, Pittsburgh, PA). Membranes were probed with mouse monoclonal antibodies (MAbs) Ab3 (1 µg/ml; Calbiochem, Darmstadt, Germany), anti-Src (1∶1000; Cell Signaling Technology, Beverly, MA), anti-phospho-Stat3 (1∶1000; Santa Cruz Biotechnology, Santa Cruz, CA), anti-actin AC-40 (0.4 mg/ml; Sigma), PoAb anti-phospho-HER2 p-Neu (tyr 1248; 0.5 mg/ml, Santa Cruz Biotechnology), anti-phospho-p44/42 MAPK (Erk1/2; 1∶2000; Cell Signaling Technology), anti-p44/42 MAPK (Erk1/2; 1∶1.000; Cell Signaling Technology), anti-phospho-Akt (Ser 473; 1∶1000; Cell Signaling Technology), anti-Akt (1∶1000; Cell Signaling Technology), anti-phospho-Src (Tyr 416; 1∶1000; Cell Signaling Technology), anti-Stat3 (1∶1000; Santa Cruz Biotechnology) and anti-E-cadherin (#4065, 1∶1000; Cell Signaling Technology).

Filters were reacted for 1 h at room temperature with HRP-conjugated goat anti-mouse Ig (1∶5000) or donkey anti-rabbit Ig (1∶10000) (Amersham GE Healthcare). Signals were detected using enhanced chemiluminescence (ECL, Amersham GE Healthcare).

### Preparation of plasma membrane extracts

The plasma membrane fraction was separated from a pool of three different cryopreserved spontaneous Δ16HER2-positive tumors lysates using the Plasma Membrane Protein Extraction Kit (Biovision, Mountain View, CA) according to the manufacturer's instructions. The plasma membrane marker E-cadherin was used in Western blot analysis to identify the plasma membrane proportion of tumor cells [19], [20].

### Morphologic and immunohistochemical analysis

Histological evaluation was done on tissue samples fixed in 10% neutral buffered formalin, embedded in paraffin, sectioned at 4 µm, and stained with hematoxylin-eosin solutions (Polysciences, Inc. Warrington, PA). For immunohistochemistry, antigen retrieval was performed in 10 mM citric acid pH 6, for 6 min at 125°C in Decloaking Chamber (Biocare Medical, Concord, CA) (c-erbB2 and PCNA) or for 20 min by continuous boiling in a microwave (luciferase). Slides were incubated for 30 min with the following primary antibodies: rabbit polyclonal anti-human c-erbB-2 oncoprotein (Dako Corp., Carpinteria, CA), mouse monoclonal anti-PCNA oncoprotein (Dako Corp.), and rabbit polyclonal anti-luciferase (Medical & Biological Laboratories Co., Naka-ku Nagoya, Japan). After incubation with the appropriate biotinylated secondary antibody, immunoreactive antigens were detected using streptavidin-peroxidase (Thermo Scientific-Lab Vision Corporation, Fremont, CA) or MACH 3 mouse HRP-polymer RtU (Biocare Medical) and DAB (Dako Corp.) or Bajoran Purple (Biocare Medical) chromogen.

### Whole-mount preparation

Whole-mount sections of all mammary glands were prepared as described [21]. Briefly, mouse skin was removed and fixed overnight in 10% buffered formalin. Mammary fat pads were scored into quarters, gently scraped from the skin and immersed in acetone overnight. After rehydration, samples were stained with ferric hematoxylin (Sigma-Aldrich), dehydrated in increasing concentrations of alcohol, cleared with histo-lemon, and stored in methyl-salicylate (Sigma-Aldrich). Digital pictures were taken with a Nikon Coolpix 995 (Nital) mounted on a stereoscopic microscope (MZ6; Leica Microsystems).

### Imaging protocol

Bioluminescence was measured noninvasively using the in VivoVision Systems, IVIS 200 Series, Imaging System for small laboratory animals (Xenogen Corp. Alameda, CA). Images were obtained 20 min after i.p. injection of luciferin as a 10-s acquisition. During image acquisition, mice were sedated continuously via inhalation of 3% isoflurane. Image analysis and bioluminescent quantification were performed using Living Image software (Xenogen Corp.).

### Statistical analysis

Statistical analysis was performed using GraphPad Prism version 5.00 for MacOSX, GraphPad Software, San Diego California USA, www.graphpad.com.

## Supporting Information

Figure S1 **In vitro validation of the bicistronic expression vector pGL3-MMTV-Δ16HER2-LUC: luciferase assay and HER2 immunodetection on transfected cells.** (A) Transient transfection studies with pGL3-MMTV-Δ16HER2-LUC in NIH3T3 and HEK293 cells, which have a high transfection rate, and in MDAMB435 cells, which present a low transfection rate, demonstrated efficient expression of luciferase, despite attenuated expression of the gene downstream from the IRES as compared with expression of the same gene placed in pGL3 control vector. Data are mean ± SEM (n = 4). (B) Expression of the MMTV-Δ16HER2-LUC cassette was verified in transfected NIH3T3 fibroblasts stained with FITC-conjugated anti-HER2 monoclonal antibody Ab3 (Oncogene), which revealed Δ16HER2 protein on the cell surface.
(TIF)

Figure S2 (A) Kaplan-Meier disease-free survival plot for F2 generation Δ16HER2-LUC transgenic mice. Note the incidence of mammary tumors and the times of tumor onset (n = 20). (B) Tumor growth curve. Data are mean ± SEM (n = 5).
(TIF)

Figure S3 **Pulmonary metastases.** Hematoxylin-eosin (left panel) and immunohistochemical staining for HER2 (middle panel) and luciferase (right panel) of intravascular lung metastases in Δ16HER2 transgenic mice. Tumor cell aggregates are strongly positive for both human HER2 and luciferase staining. Magnification: ×400.
(TIF)

Materials and Methods S1 ***In vitro*** **validation of MMTV-Δ16HER2-LUC expression and Determination of transgene insertion site.**
(DOC)
