# Supplementary figures and images for: The Human Splice Variant Δ16HER2 Induces Rapid Tumor Onset in a Reporter Transgenic Mouse
Source: PLoS One. 2011 Apr 29;6(4):e18727. doi: 10.1371/journal.pone.0018727 (PMC3084693; doi:10.1371/journal.pone.0018727)

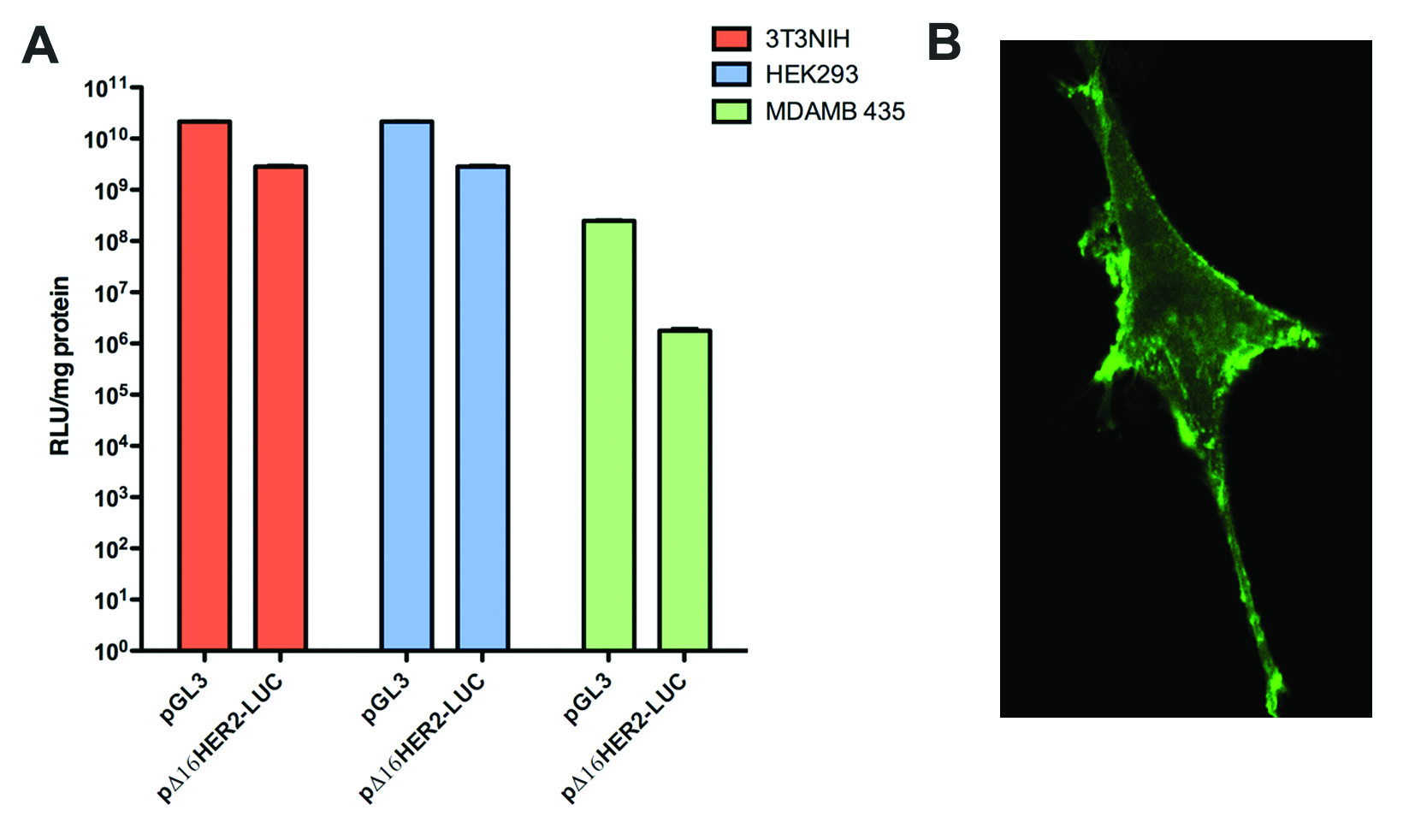

Supplement: Figure S1 — In vitro validation of the bicistronic expression vector pGL3-MMTV-Δ16HER2-LUC: luciferase assay and HER2 immunodetection on transfected cells. (A) Transient transfection studies with pGL3-MMTV-Δ16HER2-LUC in NIH3T3 and HEK293 cells, which have a high transfection rate, and in MDAMB435 cells, which present a low transfection rate, demonstrated efficient expression of luciferase, despite attenuated expression of the gene downstream from the IRES as compared with expression of the same gene placed in pGL3 control vector. Data are mean ± SEM (n = 4). (B) Expression of the MMTV-Δ16HER2-LUC cassette was verified in transfected NIH3T3 fibroblasts stained with FITC-conjugated anti-HER2 monoclonal antibody Ab3 (Oncogene), which revealed Δ16HER2 protein on the cell surface. (TIF) [file pone.0018727.s001.tif]

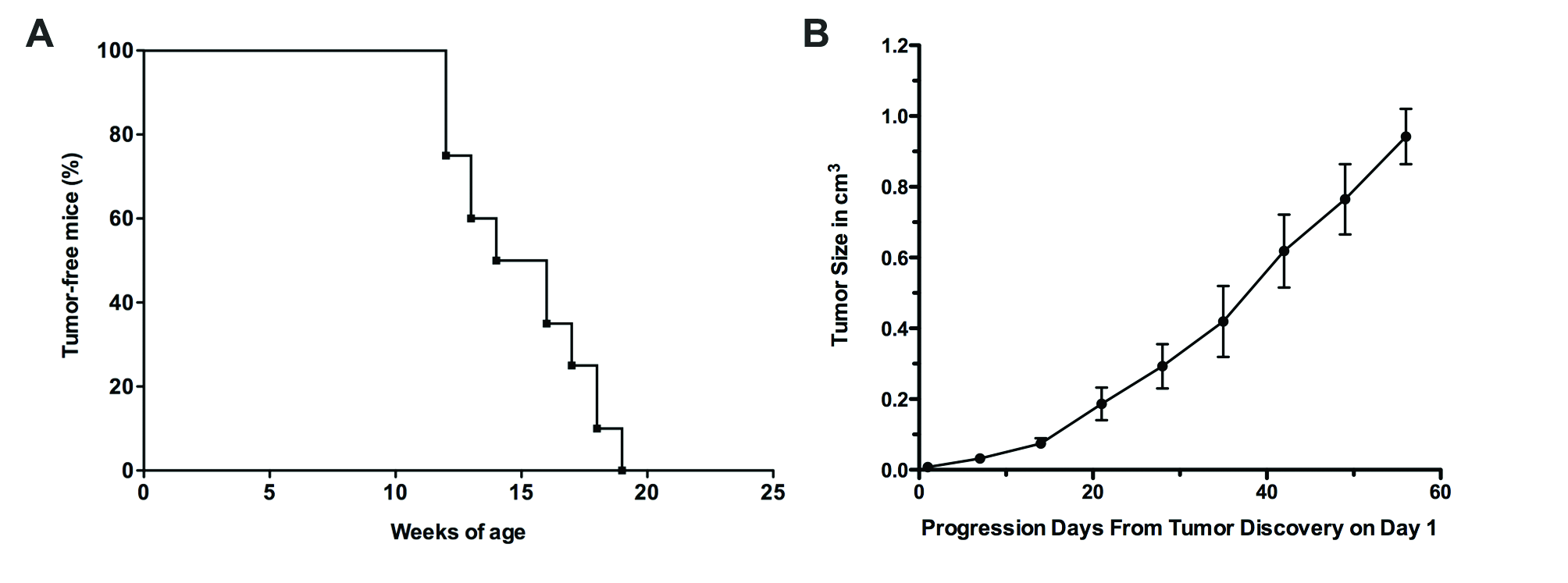

Supplement: Figure S2 — (A) Kaplan-Meier disease-free survival plot for F2 generation Δ16HER2-LUC transgenic mice. Note the incidence of mammary tumors and the times of tumor onset (n = 20). (B) Tumor growth curve. Data are mean ± SEM (n = 5). (TIF) [file pone.0018727.s002.tif]

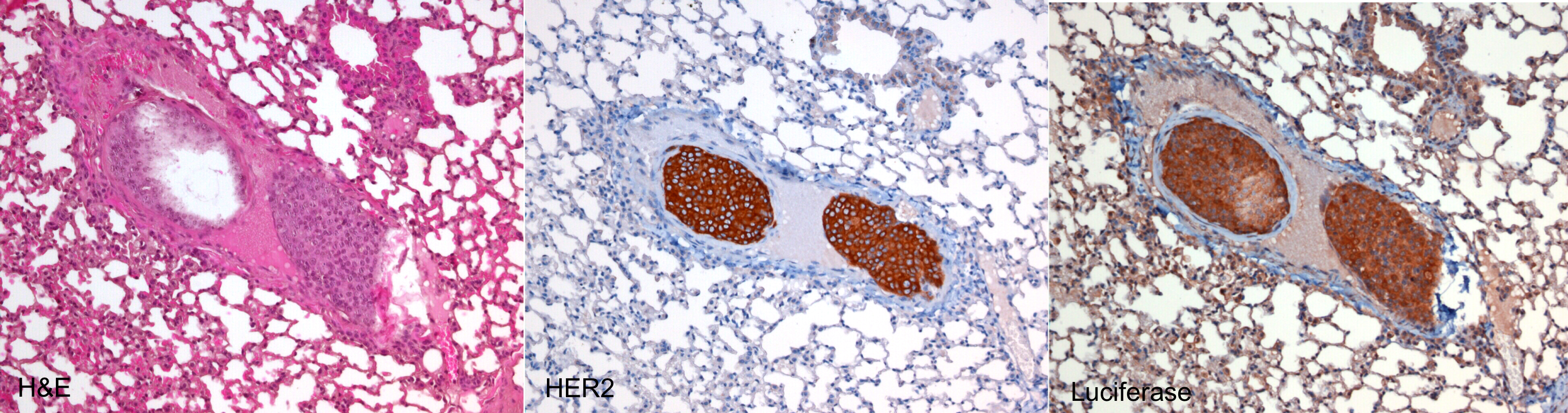

Supplement: Figure S3 — Pulmonary metastases. Hematoxylin-eosin (left panel) and immunohistochemical staining for HER2 (middle panel) and luciferase (right panel) of intravascular lung metastases in Δ16HER2 transgenic mice. Tumor cell aggregates are strongly positive for both human HER2 and luciferase staining. Magnification: ×400. (TIF) [file pone.0018727.s003.tif]
