# Supplementary material for: The Human Splice Variant Δ16HER2 Induces Rapid Tumor Onset in a Reporter Transgenic Mouse
Source: PLoS One. 2011 Apr 29;6(4):e18727. doi: 10.1371/journal.pone.0018727 (PMC3084693; doi:10.1371/journal.pone.0018727)
Supplement: Materials and Methods S1 — In vitro validation of MMTV-Δ16HER2-LUC expression and Determination of transgene insertion site. (DOC) [file pone.0018727.s004.doc]

**Supporting Information**

**Text S1. Materials and Methods**

**In vitro validation of MMTV-Δ16HER2-LUC expression.** HEK293 human embryonic kidney cells, MDAMB435 human breast adenocarcinoma cells, and NIH3T3 fibroblasts were used. Plasmid pGL3-MMTV-Δ16HER2-IRES-LUC was prepared by alkaline lysis using Endofree Qiagen Plasmid-Maxi kits (Qiagen Inc., Chatsworth, CA) according to the manufacturer's instructions and transiently transfected into cells using Lipofectamine 2000 Reagent (Invitrogen) following the manufacturer’s instructions. After 48 hours, luciferase gene expression was analyzed with the Luciferase Assay System (Promega), and light output readings were recorded on a Berthold AutoLumat luminometer LB-953 (Berthold). To verify the correct expression of Δ16HER2 protein, transfected cells were fixed in 3% paraformaldehyde, stained with FITC-conjugated c-erbB2/c-neu (Ab-3) monoclonal antibody (Oncogene), and analyzed by confocal microscopy (BIO-RAD MRC600).

**Determination of transgene insertion site.** To examine transgene integration loci, we designed three transgene-specific PCR reverse primers to the 5′ end of the transgene sequence such that each is immediately adjacent to, but not overlapping with, the previous primer. Gene-specific primer 1 is the furthest from the junction between the known transgene sequence and the unknown integration site, whereas gene-specific primer 3 is the closest. After digestion with restriction enzymes, a first round of linear PCR with gene-specific primer 1 was conducted and the product was ligated overnight at 16°C with a Y-linker previously described (16). Second and third rounds of PCR on the ligated product, performed using gene-specific reverse primer 2 and 3, respectively, and forward primers mapping on the Y-linker, yielded an amplification product containing the flanking region of the transgene, as confirmed by sequencing of the PCR product.
